# Label-Free Monitoring of Histone Acetylation Using Aptamer-Functionalized Field-Effect Transistor and Quartz Crystal Microbalance Sensors

**DOI:** 10.3390/mi11090820

**Published:** 2020-08-29

**Authors:** Tatsuro Goda, Yuji Miyahara

**Affiliations:** 1 Department of Biomedical Engineering, Faculty of Science and Engineering, Toyo University, 2100 Kujirai, Kawagoe, Saitama 350-8585, Japan; 2 Nano Innovation Institute, Inner Mongolia University for Nationalities, No. 22 HuoLinHe Street, Tongliao, Inner Mongolia 028000, China; 3 Institute of Biomaterials and Bioengineering, Tokyo Medical and Dental University (TMDU), 2-3-10 Kanda-Surugadai, Chiyoda, Tokyo 101-0062, Japan; miyahara.bsr@tmd.ac.jp

**Keywords:** post-translational modification, DNA aptamer, self-assembled monolayers, protein adsorption, potentiometry, cyclic voltammetry, chronocoulometry

## Abstract

Chemical and enzymatic modifications of amino acid residues in protein after translation contain rich information about physiological conditions and diseases. Histone acetylation/deacetylation is the essential post-translational modification by regulating gene transcription. Such qualitative changes of biomacromolecules need to be detected in point-of-care systems for an early and accurate diagnosis. However, there is no technique to aid this issue. Previously, we have applied an aptamer-functionalized field-effect transistor (FET) to the specific protein biosensing. Quantitative changes of target protein in a physiological solution have been determined by detecting innate charges of captured protein at the gate-solution interface. Moreover, we have succeeded in developing an integrated system of FET and quartz crystal microbalance (QCM) sensors for determining the adsorbed mass and charge, simultaneously or in parallel. Prompted by this, in this study, we developed a new label-free method for detecting histone acetylation using FET and QCM sensors. The loss of positive charge of lysine residue by chemically induced acetylation of histone subunits (H3 and H4) was successfully detected by potentiometric signals using anti-histone aptamer-functionalized FET. The adsorbed mass was determined by the same anti-histone aptamer-functionalized QCM. From these results, the degree of acetylation was correlated to the charge-to-mass ratio of histone subunits. The histone required for the detection was below 100 nM, owing to the high sensitivity of aptamer-functionalized FET and QCM sensors. These findings will guide us to a new way of measuring post-translational modification of protein in a decentralized manner for an early and accurate diagnosis.

## 1. Introduction

In recent years, the highly sensitive detection of disease-related systemic biomolecules, called biomarkers, has gained considerable attention for the early detection and treatment of cancer and diseases [1,2]. Most of the conventional biosensing techniques quantitatively measure the level of gene expression and free protein in body fluids. However, a quantitative change of a single biomolecule provides limited information about the physiological conditions of an individual. Diagnosis of complicated living systems from one aspect always contains the risk of false-positive/negative results. A comprehensive analysis using various aspects such as genomics, gene expression, transcriptional regulation, protein translation, and post-translational modification in cost-effective ways may realize accurate diagnosis in early stages [3]. Qualitative changes in biomolecules such as post-translational modification of protein are closely related to the progression of diseases, as represented by phosphorylation of the tau protein in Alzheimer’s diseases [4,5]. Chemical modifications of amino acid residues, including histone acetylation and methylation, play pivotal roles in gene transcriptional regulation [6,7]. These post-translational modifications are closely related to epigenomics. Therefore, it is essential to elucidate oncogenic mechanisms caused by transcriptional dysregulations [8,9].

Histone is a cylindrical cationic protein containing 20% or more of strong basic amino acid residues and wraps around a long anionic DNA by electrostatic forces [10]. The histone/DNA complex, nucleosome, is the basic unit of DNA packaging in the cell nucleus. Histone forms an octamer by collecting two molecules of each of the four types of subunits, H2A, H2B, H3, and H4 [11]. One histone octamer assembles a nucleosome by wrapping around 146 base pairs of DNA duplex counterclockwise, for about 1.65 laps. Histone acetylation occurs by histone acetyltransferase (HAT) to the lysine (Lys) residues (–NH–Ac or –N–Ac_2_) at the site called the histone tail [12]. The chemical modification triggers the DNA dissociation by weakening electrostatic attractions to promote DNA transcription. Also, histone is reversibly deacetylated (–NH_3_^+^) by histone deacetylase (HDAC) [13]. Moreover, histone undergoes phosphorylation, methylation, and ubiquitination [14]. It has been proven that these chemical modifications are involved in regulating various chromatin functions, including gene expression. The dysfunction of DNA transcription caused by the irregular post-translational modifications is closely related to carcinogenesis [15].

Post-translational modification has been extensively analyzed in basic biology. Chemical modifications of amino acid residues are mainly analyzed by mass spectrometry [16]. Post-translational modifications in chromatin are studied by immunoprecipitation, followed by next-generation sequencing [17]. However, existing analytical methods have drawbacks, such as analysis time, cost, and throughput. Mass spectrometry and next-generation sequencing are powerful but require long analysis time, specialized facilities, and technicians. Immunoprecipitation requires a complicated procedure, such as blocking, recognition reaction, primary/secondary antibody labeling, spectroscopic detection, and optical settings. These measurements are incompatible with point-of-care testing.

**Figure 1:**
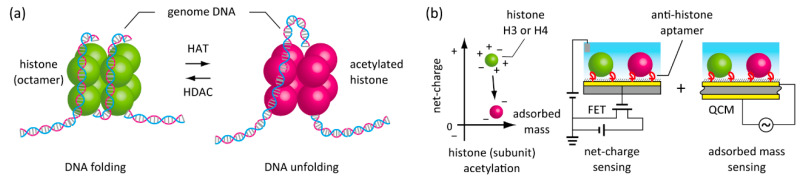
Schematics showing in situ label-free detection of histone acetylation. (**a**) Enzymatic acetylation and deacetylation of histone in the cell nucleus for the regulation of gene transcription. (**b**) A new strategy to detect histone acetylation through the measurements of net-charge and adsorbed mass using aptamer-functionalized field-effect transistor (FET) and quartz crystal microbalance (QCM) biosensors in parallel.

In this study, we propose a new method for label-free detection of post-translational modification by charge density changes of protein molecules using a potentiometric biosensor (Figure 1). A field-effect transistor (FET) can transduce biorecognition events on the gate dielectric into the potentiometric signal [18,19]. The device can measure ions and innate biomolecular charges in the solution in contact with the gate dielectric or the extended gate by the field effect. The bio-transistors have found applications for nucleic acid sequencing [20,21], glycan determination on the cell surface [22,23], and biomarker detections [24,25]. Although protein is weak in charge density and much bigger than the Debye length of the physiological solution, we have succeeded in the label-free detection of protein adsorbed on the FET sensor surface [26], because the biosensor detects local charges on the protein on the gate dielectric even under the physiological electrolytic conditions [27,28]. Moreover, specific recognition of protein was successful using a DNA aptamer-functionalized FET [25,29]. Importantly, a FET sensor was able to incorporate into a quartz crystal microbalance (QCM) sensor as another label-free sensor [30]. The FET/QCM-integrated device simultaneously determined the charge and mass of the captured target molecule in situ without labeling. The bimodal analysis provided information about nanostructural alterations of protein adsorption over time. In this study, we applied the charge and mass detection strategy to analyze the post-translational modification of proteins. For the proof-of-concept study, chemically induced histone acetylation was applied to the FET and QCM sensing in parallel. Attempts to detect post-translational modifications from the changes in protein charge and adsorbed mass can expand the applicability of FET biosensors. The achievement will allow genome, epigenome, and proteome analysis on a semiconductor device as a common sensing platform, with rapid, inexpensive, parallel, and miniature features, toward an early accurate diagnosis of cancer or diseases.

## 2. Materials and Methods

### 2.1. Materials

We used recombinant human histone H3 (H3.1) and H4 obtained from New England BioLabs, Japan (Tokyo, Japan), sulfo-*N*-hydroxysulfosuccinimide acetate (sulfo-NHS-acetate) from Thermo Fisher Scientific, Japan (Tokyo, Japan), fluorescamine from Funakoshi (Tokyo, Japan), sulfobetaine-3-undecanethiol (SB) from Dojindo (Kumamoto, Japan), and synthetic oligo-DNA aptamers with high performance liquid chromatography (HPLC) purification grade from Tsukuba Oligo Service (Tsukuba, Ibaraki, Japan). Hexamine-ruthenium (III) chloride (RuHex), tris(2-carboxyethyl)phosphine), 11-mercapto-1-undecanol, and 11-mercaptoundecyl phosphoric acid were purchased from Sigma-Aldrich, Japan (Tokyo, Japan). All the reagents were used without further purification. Deionized pure water (18.2 MΩ·cm^−1^, 0.22 μm-filtration, Millipore, Bedford, MA, USA) was used for all experiments.

### 2.2. Histone Acetylation

20 mmol/L sulfo-NHS-acetate aqueous solution, 1 mg/mL histone H3 or H4 in 0.1 mol/L sodium carbonate buffer (pH 8.5), and water were mixed to give an 82 µL solution at the desired molar ratio of sulfo-NHS-acetate and Lys residues. The reaction proceeded for 1 h at room temperature.

Acetylated histone H3 and H4 were analyzed by liquid chromatograph-mass spectrometry (LC-MS) using a maXis4G-CPR system (Bruker Daltonics, Billerica, MA, USA). Samples were desalted by filtration prior to the measurements.

The fluorescamine assay quantified free Lys content. 2 µL of histone H3 (0.49 mg/mL) or H4 (0.45 mg/mL), 10 µL of 2% trimethylamine in dimethyl sulfoxide, and 5 mg/mL of fluorescamine in *N,N*-dimethylformamide were mixed for 10 min. Then, the fluorescence intensity of the solution was measured at Ex/Em = 365/470 nm/nm using a Nanodrop ND 3300 spectrophotometer (Thermo Fisher Scientific Japan).

### 2.3. Electrode Functionalization

A planar gold electrode was cleaned prior to use with 1 mol/L NaOH and 1 mol/L HCl. After drying, it was soaked in a 1× Dulbecco’s phosphate-buffered saline (DPBS) solution containing 5 µmol/L anti-histone aptamer, 50 µmol/L tris(2-carboxyethyl)phosphine), and 0.1 mol/L KCl overnight at room temperature. After the reaction, the electrode was washed with water and dried. Then, the aptamer-functionalized electrode was immersed in 10 mmol/L SB in DPBS overnight. After the reaction, the electrode was washed with water and dried until use.

### 2.4. Electrochemistry

The lateral density of anti-histone DNA aptamer on the functionalized gold electrode was determined by chronocoulometry using an Autolab PGSTAT 302 potentiostat (Eco Chemie, Utrecht, The Netherlands) equipped with a three-electrode system using a platinum wire as a counter electrode and an Ag/AgCl electrode in saturated KCl solution via a salt bridge as a reference electrode. Chronocoulometry was performed at a pulse period of 1000 ms, and the potential stepped from 125 to −300 mV (vs. reference electrode) in 15 mmol/L DPBS with/without 50 μmol/L RuHex [25,29].

The lateral density of SB self-assembled monolayer (SAM) was determined by cyclic voltammetry using an Autolab PGSTAT 302 potentiostat using the three-electrode system. The aptamer/SB SAM-functionalized gold electrode as a working electrode was soaked in degassed 0.5 M KOH in 3.3 M KCl. The potential was scanned three times from −0.1 to −1.3 V (vs. reference electrode) at the scan rate of 0.5 V/s [26,30].

### 2.5. FET Sensing

Ten round-shape gold electrodes (500 μm in diameter) on a glass-epoxy resin chip (Towa Tech, Shizuoka, Japan) were used after functionalization with the aptamer and SB SAM. Each electrode was connected to the FET gate in a Keithley 6517B electrometer/high-resistance meter (Keithley, Cleveland, OH) through a switching circuit. An Ag/AgCl electrode in a saturated KCl solution via a salt bridge was used as a reference electrode. We used a measurement buffer containing 1 mmol/L phosphate and 140 mmol/L KCl (pH 7.4) for the binding experiments. First, the aptamer/SAM-modified gold electrode was annealed in the measurement solution at 100 °C, and then slowly cooled down to room temperature prior to the measurements. Then, the gate potential was started to record. The working and reference electrodes were kept at 25 °C during the measurements. After stabilization of the gate potential for at least 1 h, the binding experiment was conducted in the histone solutions with sequential increases in the target protein concentration (33, 97, 221, 457, 975, and 1950 nmol/L for histone H3, and 44, 132, 301, 621, 1330, and 2650 nmol/L for histone H4) every 10 min. 

### 2.6. QCM Sensing

The QCM measurements were performed on the aptamer/SAM-functionalized Au on a piezoelectric quartz sensor at the fundamental frequency of 30 MHz using a NAPICOS QCM analyzer (Nihon Dempa Kogyo, Tokyo Japan) at 25.00 ± 0.02 °C. The surface-immobilized aptamer was annealed prior to the measurement. The binding experiment was performed by monitoring the resonance frequency change in a histone H3 or H4 solution with sequential increases in the target protein concentration (same as the FET measurements) by flushing 200 μL protein solutions using a syringe pump at a flow rate of 3 mL/h.

## 3. Results

### 3.1. Lys Acetylation

**Figure 2:**
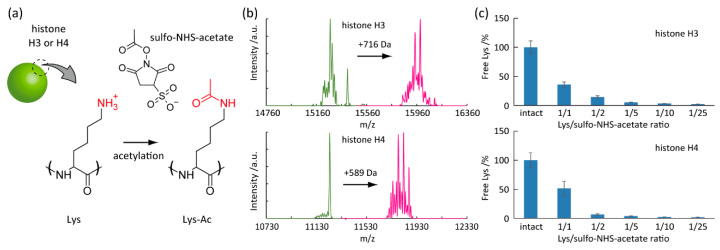
Model studies of histone acetylation. (**a**) Acetylation of Lys in histone subunit (H3 or H4) induced by sulfo-*N*-hydroxysulfosuccinimide acetate (sulfo-NHS-acetate). (**b**) Liquid chromatograph-mass spectrometry (LC-MS) analysis of histone H3 and H4 before and after treating sulfo-NHS-acetate. (**c**) Free Lys degree in histone H3 and H4 as a function of sulfo-NHS-acetate content.

For the proof-of-concept study of label-free detection of histone acetylation, we chemically induced the Lys-selective acetylation in histone H3 and H4 subunits by sulfo-NHS-acetate (Figure 2a) [31]. Histone H3 and H4 comprise 136 and 103 amino acids and contain 13 and 11 Lys residues, respectively [32,33]. Lys acetylation converts the positive charge of the primary amines with pKa 10.3 into neutral. As a result, the protein net-charge shifts to the negative direction. The protein calculator v3.4 predicts that the net-charge estimated by the amino acid sequence significantly drops from 20.3 to 7.4 at pH 7.0 [34]. Similarly, the complete Lys acetylation in histone H4 impairs the net-positive charges from 18.4 to 7.4 at pH 7.0. The Lys acetylation was confirmed by the gain of protein mass using LC-MS (Figure 2b). The acetylation by sulfo-NHS-acetate increased m/z (mass per charge) by 716 and 589 units for histone H3 and H4, respectively. The mass increases correspond to 17 and 14 acetyl groups, respectively. Therefore, all Lys residues could be chemically acetylated (–NH–Ac or –N–Ac_2_) by sulfo-NHS-acetate. Besides, the percentage of free Lys residue was determined by the fluorescamine assay (Figure 2c). We found that the degree of acetylation was tunable by the molar ratio of Lys residue and sulfo-NHS-acetate in the reaction mixture. We used the chemically acetylated histone H3 and H4 for the subsequent experiments.

### 3.2. Electrode Characterization

**Figure 3:**
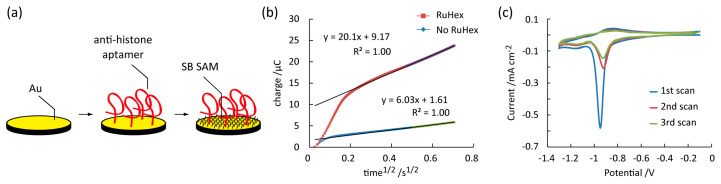
Surface functionalization of gold electrodes for specific recognition of histones. (**a**) Schematics showing a two-step introduction of anti-histone single-stranded DNA aptamers and sulfobetaine-3-undecanethiol (SB) self-assembled monolayer (SAM) on a planar gold electrode. (**b**) Chronocoulometry with and without hexamine-ruthenium (III) chloride (RuHex) for determining the surface density of the aptamer. (**c**) Cyclic voltammetry for determining the surface density of SB SAM.

We covalently introduced anti-histone aptamers composed of single-stranded DNA with a 6-mercaptohexyl linker in the 5′ end on a planar gold electrode for sensitive and specific recognition of the histone subunits (Figure 3a). The anti-histone H3 and H4 aptamer sequences were: 5′-TTT GAG TGT GGT TCC CGG GAG GGC GCC TAC GGG TCC CGT ATT CGG ATT TGT GC-3′ (53 mer) and 5′-TTT TGG TGG GGT TCC CGG GAG GGC GGC TAC GGG TTC CGT AAT CAG ATT TGT GT-3′ (53 mer), respectively [35]. These aptamers form a G-quadruplex conformation with potassium ions for recognizing each histone subunit. Then, the remaining surface was backfilled by anti-fouling SB SAM for preventing nonspecific adsorption of protein on the electrode [36,37]. By doing so, we have previously succeeded in detecting target protein using aptamer-functionalized electrodes in realistic dirty samples.

Chronocoulometry was performed to determine the lateral aptamer density on the modified electrode (Figure 3b). The difference in the *y*-intercept in the linear fit (Δ*Q_CC_*) represents the redox charge per unit area from RuHex^3+/2+^ that is stoichiometrically bound on the DNA aptamer [25,29]. Therefore, the aptamer density (*Γ_apt_*) was determined by the following equation:

*Γ_apt_* = (Δ*Q_CC_N_A_*/*F*) (*m*/*n*), (1)

where *N_A_*, *F*, and *n/m* represent the Avogadro’s number, Faraday’s constant, and the charge ratio between RuHex^3+^ and single DNA aptamer chain (*n/m* = 3/53), respectively. An average *Γ_apt_* was 0.0205 ± 0.0099 and 0.0211 ± 0.0048 chain/nm^2^ for anti-histone H3 and anti-histone H4 aptamers. Therefore, the lateral aptamer distance (1/*Γ_apt_^0.5^*) was calculated to be 7.4 ± 1.5 and 7.0 ± 0.9 nm. The relatively large distances account for the elasticity theory for a polymer in good solvents. The excluded volume effect for the aptamer dominates the lateral distance by a scaling law of the radius of gyration (*R_g_*) in good solvents (*R_g_*~*M_w_^n^*) [38,39]. The electrostatic repulsive forces also limit the aptamer crowding on the electrode surface. On the other hand, these effects are advantageous for a surface-immobilized aptamer to secure the proper space required for capturing a bulky target protein.

The surface density of SAM (*Γ_SAM_*) was characterized by cyclic voltammetry (Figure 3c) [26,30]. The peak area in the first negative scan (Δ*Q_SAM_*) represents the reducing charge per unit area of the thiol group in SB from the gold electrode, as follows:

*Γ_SAM_* = Δ*Q_SAM_N_A_*/*F*, (2)

The SAM density was calculated to be 5.3 ± 0.1 and 5.1 ± 0.1 chains/nm^2^ for anti-histone H3/SB SAM and anti-histone H4/SB SAM, respectively. These values were in agreement with the theoretical value of alkanethiol SAM and our previous SB SAM results. Dramatic decreases in Δ*Q_SAM_* in the second and third scans stand for the irreversible breakdown of SAM structure by chemically desorbing from the gold electrode after the first scan. The lateral distance (1/*Γ_SAM_^0.5^*) was 4.3–4.5 Å, which is an order of magnitude smaller than the protein size (typically >5 nm). Therefore, the SB SAM was densely aligned on the surface via hydrophobic interaction between the alkyl groups [40]. The SAM layer prevented nonspecific adsorption of histone H3 and H4 on the gold electrode by separating each other. SB is especially known to repel protein by forming a thick hydration layer around the zwitterionic sulfobetaine moiety [36,37].

### 3.3. Detection of Histone Acetylation

**Figure 4:**
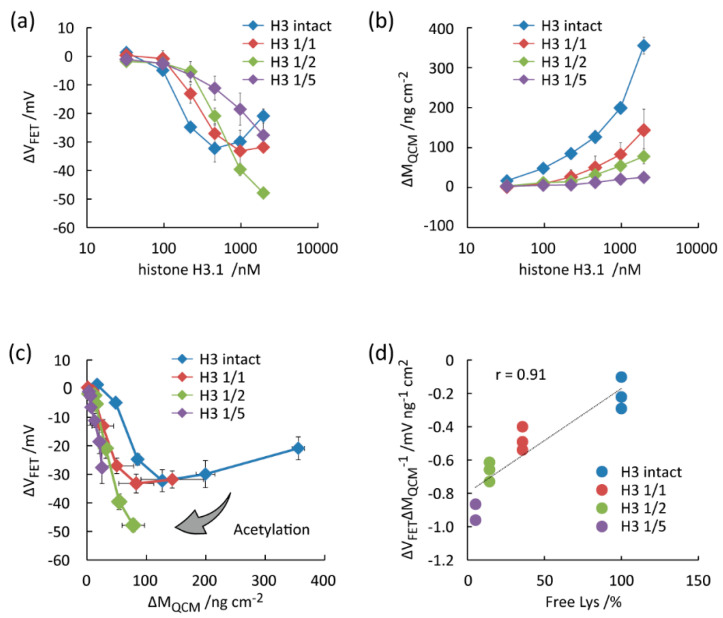
Evaluation of the degree of Lys acetylation in histone H3 treated with sulfo-NHS-acetate through parallel measurements of FET and QCM using the gold electrode functionalized by anti-histone H3 aptamer and SB SAM. (**a**) Δ*V_FET_* vs. histone H3.1 concentration. (**b**) Δ*M_QCM_* vs. histone H3.1 concentration. (**c**) Δ*V_FET_* vs. Δ*M_QCM_*. (**d**) Δ*V_FET_*/Δ*M_QCM_* vs. free Lys contents in histone H3.1.

With the successful chemical acetylation of Lys residues in histone and the aptamer functionalization of sensing electrode in hands, we determined the degree of histone acetylation from interface potential and adsorbed mass using FET and QCM sensors. The net-charge of histone H3 at varying degrees of Lys acetylation was determined by capturing the anti-histone H3 aptamer present on the extended gate FET sensor (Figure 4a). The potential difference from the no histone H3 condition (Δ*V_FET_*) is proportional to the net-charges per unit area of histone H3 adsorbed on the FET sensor (Δ*Q_FET_*), as shown in Equation (3) [25,26,28,30].

Δ*V_FET_* = Δ*Q_FET_*/*C_DL_* = *qΓ_protein_*/*C_DL_*, (3)

where *C_int_, q,* and *Γ_protein_* represent the electrical double-layer capacitance per unit area, net-charge of a single protein, and surface density of protein, respectively. Therefore, Δ*V_FET_* is proportional to *Γ_protein_* when *q* and *C_DL_* are constant. In reality, *C_DL_* varies before and after protein adsorption, depending on the protein type, conformation, orientation, and thin organic layer on the electrode. In such cases, Δ*V_FET_* is negative even after the adsorption of a positively charged protein [26,28]. The binding of pristine histone H3 to the aptamer-immobilized FET generated negative Δ*V_FET_* at 0 to 460 nmol/L histone H3. Further increases in histone H3 concentration of the measurement solution caused a Δ*V_FET_* recovery in a positive direction. A trend was similar in acetylated histone H3 modified by sulfo-NHS-acetate with the molar Lys/sulfo-NHS-acetate ratio of 1/1 (abbreviated as “H3 1/1”). Δ*V_FET_* decreased by the concentration for “H3 1/2” and “H3 1/5”.

We performed QCM measurements using the same aptamer-functionalized electrode and the same protein concentrations to estimate the adsorbed mass of protein per unit area (Δ*M_QCM_*) (Figure 4b). Because we focus on the *q* variation as a function of the acetylation degree of histone by comparing FET and QCM results, the change in a resonator frequency from the one at the no histone H3 condition (Δ*f*) is correlated to Δ*M_QCM_* when a small elastic mass is added to the crystal surface [41]:

Δ*M_QCM_* = *pΓ_protein_* = *s*Δ*f*, (4)

where *p*, *Γ_protein_*, and *s* represent the molar mass of histone H3, adsorbed number of histone H3 per unit area, and Sauerbray constant (−0.46 ng/cm^2^ Hz), respectively. Although Δ*f* cannot rigorously determine the adsorbed mass in liquid-phase measurements due to energy dissipating factors, QCM semi-quantifies the amount of histone captured. Δ*M_QCM_* monotonically increased by histone H3 concentration in the measurement solutions [41]. On the other hand, Δ*M_QCM_* decreased by the degree of acetylation at the same concentrations, implying that the acetylated histones impair the binding ability to the anti-histone aptamer on the surface.

Using the FET and QCM results, a scatter plot was obtained for histone H3 at the varying degree of acetylation (Figure 4c). Polygonal lines were formed in the scatter plot for “H3 intact” and “H3 1/1”. The results were presumed by the *C_DL_* changes following protein adsorption. The polygonal lines rotated clockwise around the origin by increasing the degree of acetylation (from “H3 intact” to “H3 1/5”). The trend is explained by the loss of the positive charge of histone [27]. In fact, from Equations (3) and (4), the net-charge of a single protein (*q*) can be evaluated by the slope value of the scatter plot (Δ*V_FET_/*Δ*M_QCM_*), as follows:

Δ*V_FET_*/Δ*M_QCM_* = *q*/*pC_DL_*. (5)

By assuming *p* and *C_DL_* as constant, the slope value decreases when histone H3 loses positive charges by Lys acetylation. Namely, the degree of histone acetylation can be estimated by the slope value of Δ*V_FET_/*Δ*M_QCM_*. The Δ*V_FET_*/Δ*M_QCM_* values were well separated with the different acetylation degrees at 97–457 nmol/L histone H3 and 221–1950 nmol/L acetylated histone H3 (Figure 4d). The correlation coefficient was 0.91.

**Figure 5:**
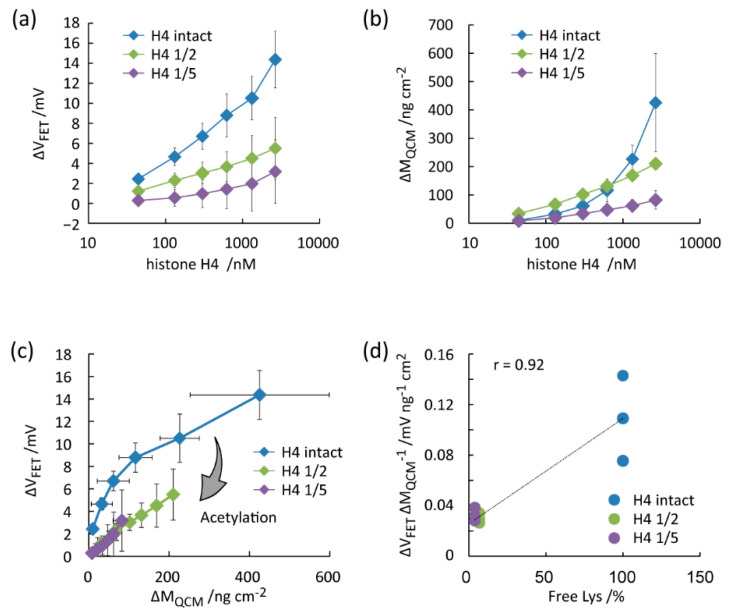
Evaluation of the degree of Lys acetylation in histone H4 treated with sulfo-NHS-acetate through parallel measurements of FET and QCM using the gold electrode functionalized by the anti-histone H4 aptamer and SB SAM. (**a**) Δ*V_FET_* vs. histone H4 concentration. (**b**) Δ*M_QCM_* vs. histone H4 concentration. (**c**) Δ*V_FET_* vs. Δ*M_QCM_*. (**d**) Δ*V_FET_*/Δ*M_QCM_* vs. free Lys contents in histone H4.

FET and QCM measurements were also performed for intact histone H4 and its acetylated forms (Figure 5a,b). Differently from histone H3, Δ*V_FET_* and Δ*M_QCM_* monotonically increased by the histone H4 concentrations for all samples. Δ*V_FET_* was the highest for “H4 intact”, followed by “H4 1/2” and “H4 1/5”. The results suggest that the positive charges of histone H4 were lost by the acetylation of Lys residues [27]. For validation, the net-charge of a single protein (*q*) was estimated in the scatter plot (Figure 5c). In agreement with the histone H3 results, the slope value of the polygonal lines decreased by the acetylation degree. The Δ*V_FET_/*Δ*M_QCM_* decreased by decreasing the free Lys contents with the correlation coefficient of 0.92 (Figure 5d). The correlation coefficient was 0.92. The slope value was the highest for “H4 intact” at 0 to 300 nmol/L. The slope value was almost the same between “H4 1/2” and “H4 1/5”. The trend is explained by the free Lys content, as shown in Figure 2c and Figure 5d. The Δ*V_FET_*/Δ*M_QCM_* values were distinguishable at 44 nmol/L histone H4 with different degrees of acetylation. The relatively low detection limit is attributed to the high affinity of the anti-histone H4 aptamer to the target [35]. Therefore, we concluded that the degree of Lys acetylation in histone H3 and H4 were successfully estimated by combining FET and QCM measurements using the aptamer/SB SAM-functionalized gold electrodes.

## 4. Discussion

A designer interface is essential for monitoring histone acetylation by FET and QCM sensors. Selective binding of histone H3 and H4 to their corresponding aptamers on the electrodes in an orientation-controlled manner is one of the successful reasons for the quantification of acetylation-induced charge-conversion of Lys residues using FET sensors [25,26,28]. Local charges of protein out of the electrical double layer cannot be detected due to the screening effect by mobile counter ions in an electrolyte solution. Thus, FET-based sensors typically detect charges of protein limited in the Debye length from the surface. Since the charges of amino acid residues are heterogeneously distributed on the surface of protein as a colloidal macromolecule, the potentiometric signal of FET heavily depends on the orientation and conformation of the protein adsorbed on the electrode as well as the amount adsorbed [28]. Since nonspecifically adsorbed proteins are randomly oriented on the surface, it is hard to determine the site-selective charge-conversion of Lys residues. In fact, we were unable to determine the Lys acetylation of histone H3 and H4 using electrodes functionalized by 11-mercapto-1-undecanol or 11-mercaptoundecyl phosphoric acid SAMs without the aptamers (data not shown).

One of the challenges of our proposed system is that the anti-histone aptamers dramatically lose the binding affinity to the acetylated histones. Therefore, the binding events are hampered even at the high protein concentrations (Figure 4 and Figure 5). To solve this, affinity ligands that can capture histone irrespective of the degree of acetylation are need.

## 5. Conclusions

We have developed a new method for evaluating the degree of histone acetylation as an essential post-translational modification of protein for gene transcription. Chemically induced acetylation of Lys residues was detected by the changes in net-charge and mass of histone H3 and H4 bound on the anti-histone aptamer/SB SAM-functionalized gold electrode using FET and QCM sensors. The anti-histone aptamers allowed to capture histone at fixed orientation to the electrode that helped compare net-charges of histone at different acetylation degrees. The anti-histone aptamers also contributed to capturing the targets below 100 nM. At the same time, the anti-fouling SB SAM layer prevented nonspecific adsorption of histone on the surface. As a proof-of-concept study, the ratio between the potential change in FET measurement and the mass change in QCM measurements decreased by increasing the degree of acetylation because of the loss of positive net-charges of Lys residues in histone subunits. Our study is the first achievement of in situ detection of a qualitative change of protein using the dual label-free sensors in parallel.
